# Supplementary material for: Unprecedented decline of Arctic sea ice outflow in 2018
Source: Nat Commun. 2022 Apr 1;13:1747. doi: 10.1038/s41467-022-29470-7 (PMC8975830; doi:10.1038/s41467-022-29470-7)
Supplement: Supplementary file 1 — Supplementary Information [file 41467_2022_29470_MOESM1_ESM.pdf]

Supplementary information for

Unprecedented decline of Arctic sea ice outflow in 2018

Hiroshi Sumata[1*], Laura de Steur[1], Sebastian Gerland[1], Dmitry V. Divine[1], Olga Pavlova[1]

[1]Norwegian Polar Institute, Fram Centre, Tromsø, Norway

* Correspondence to: Hiroshi Sumata (hiroshi.sumata@npolar.no)

Contents of this supplementary information

Supplementary Tables S1 to S4.
Supplementary Figures S1 to S14.

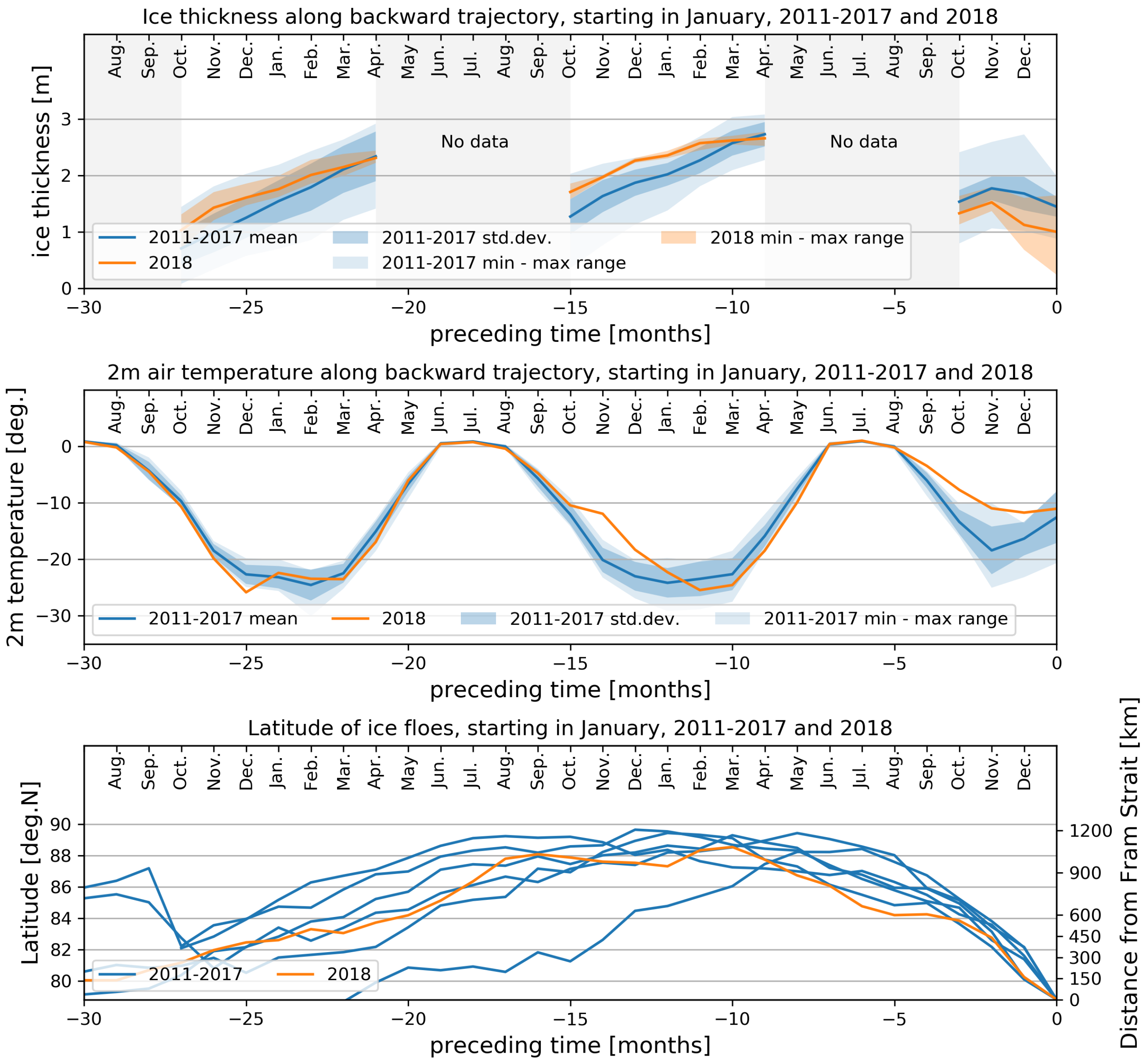

Figure S1. The same with Figure 5b, c, and d, but for reconstructions for ice floes arrived at Fram Strait in January, 2011 – 2017 and 2018.

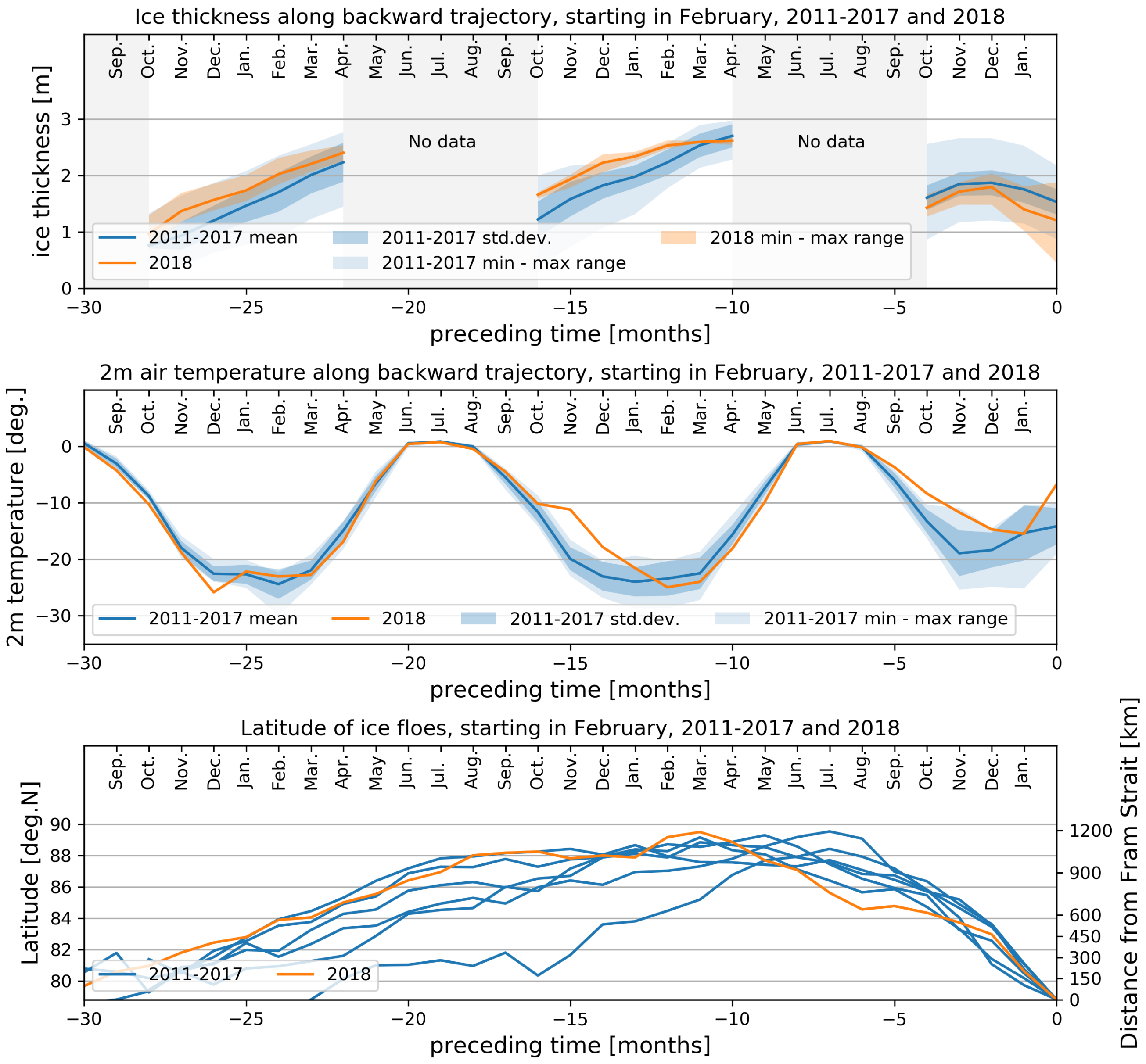

Figure S2. The same with Figure 5b, c, and d, but for reconstructions for ice floes arrived at Fram Strait in February, 2011 – 2017 and 2018.

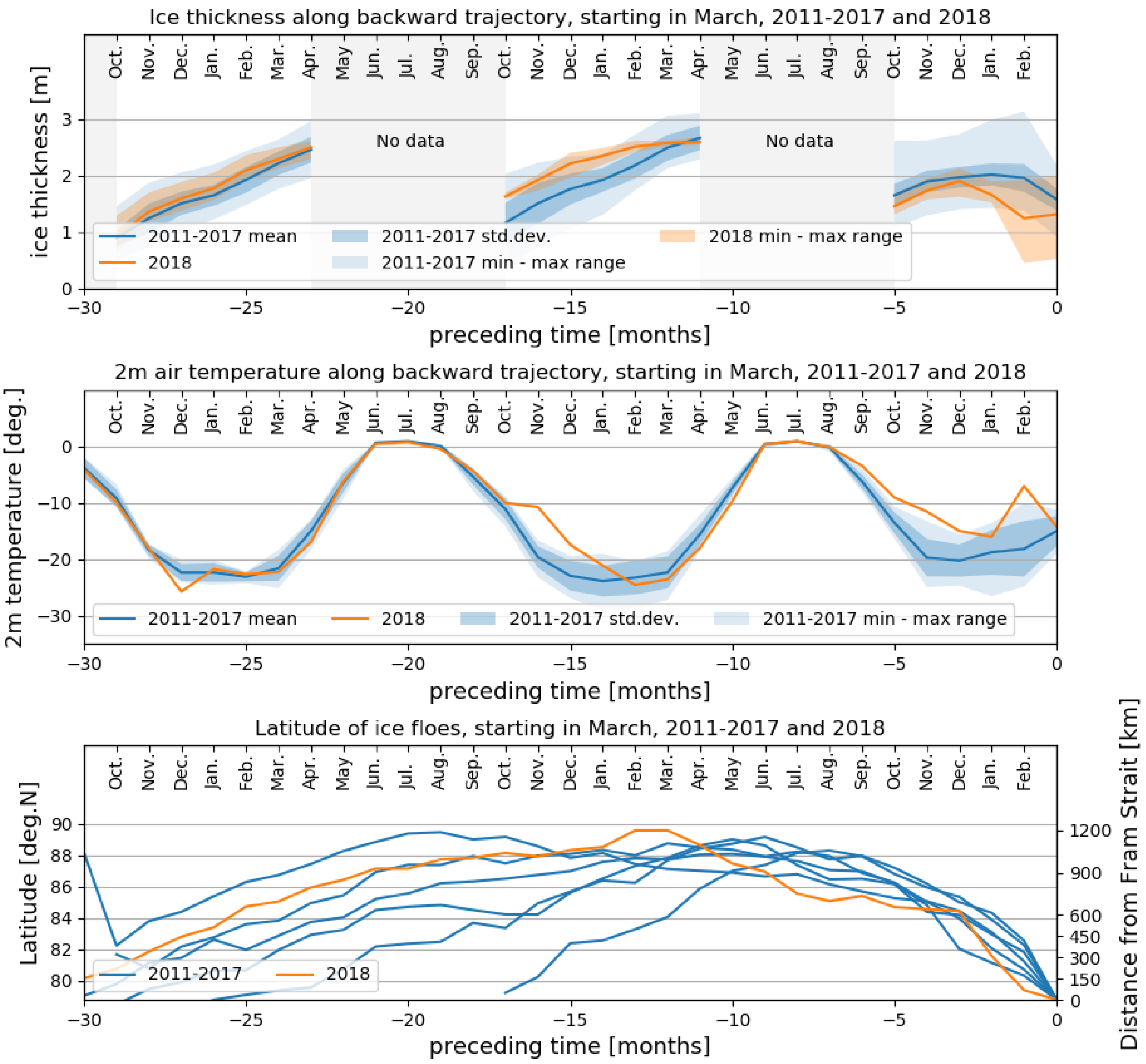

Figure S3. The same with Figure 5b, c, and d, but for reconstructions for ice floes arrived at Fram Strait in March, 2011 – 2017 and 2018.

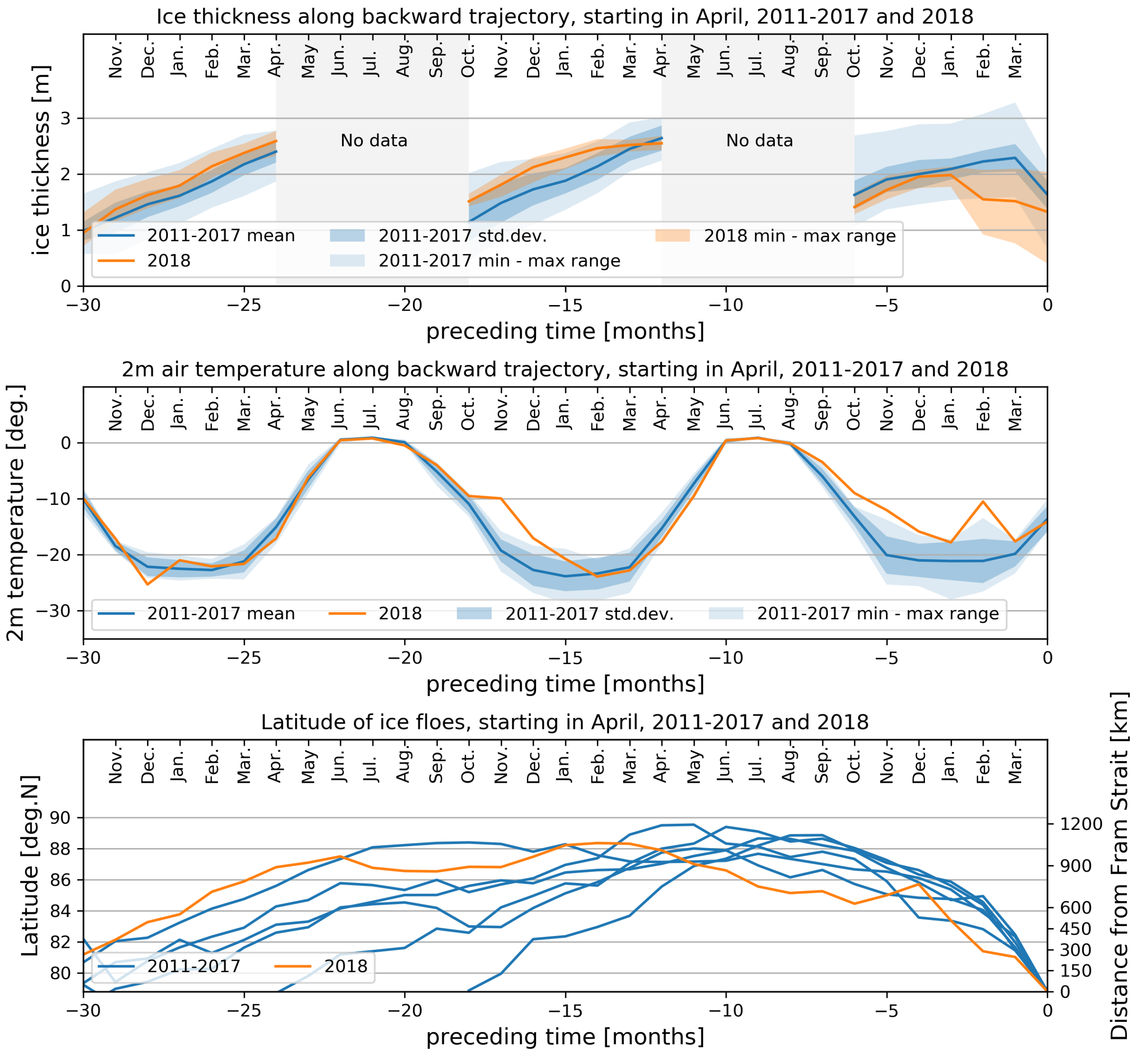

Figure S4. The same with Figure 5b, c, and d, but for reconstructions for ice floes arrived at Fram Strait in April, 2011 – 2017 and 2018.

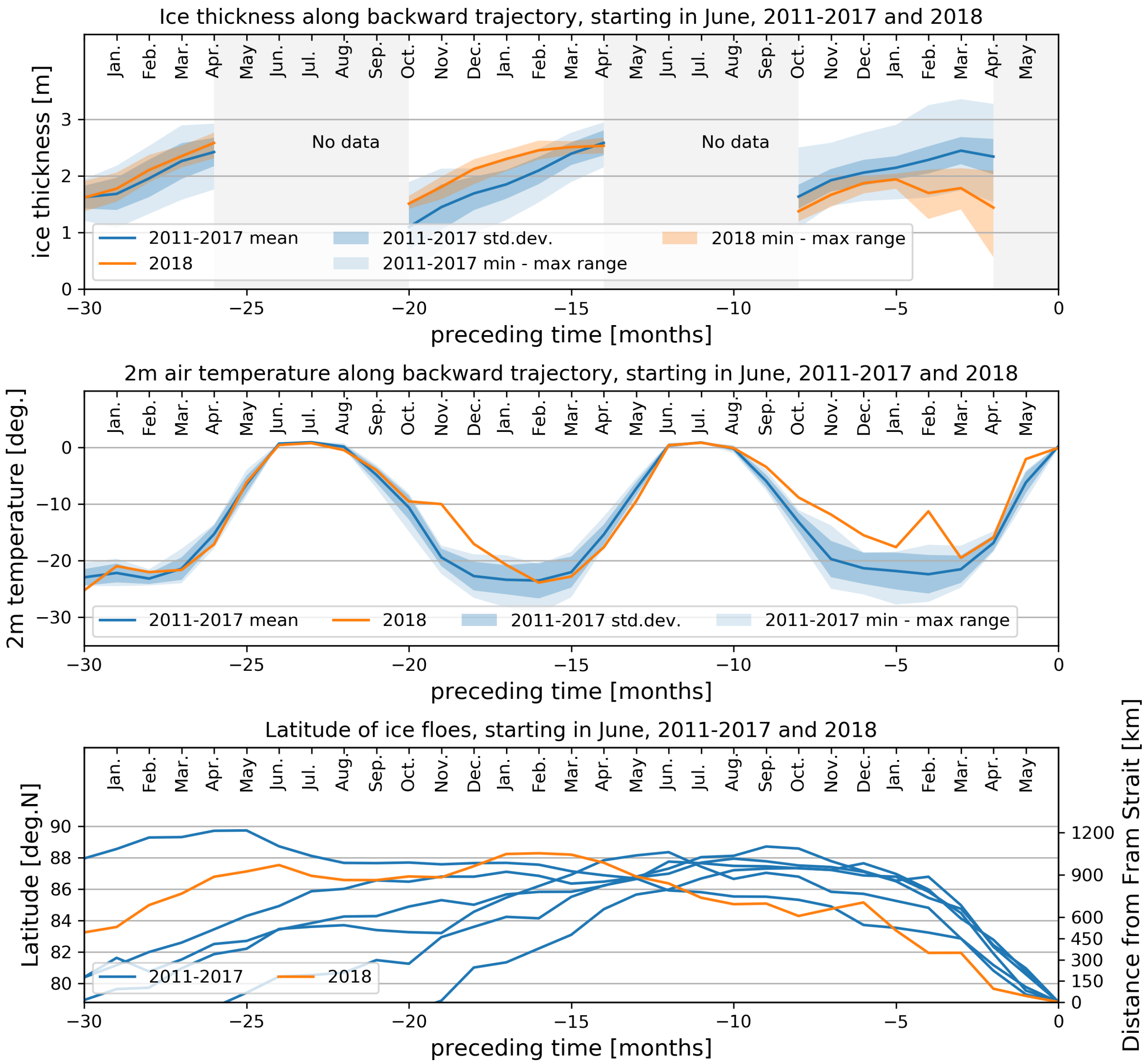

Figure S5. The same with Figure 5b, c, and d, but for reconstructions for ice floes arrived at Fram Strait in June, 2011 – 2017 and 2018.

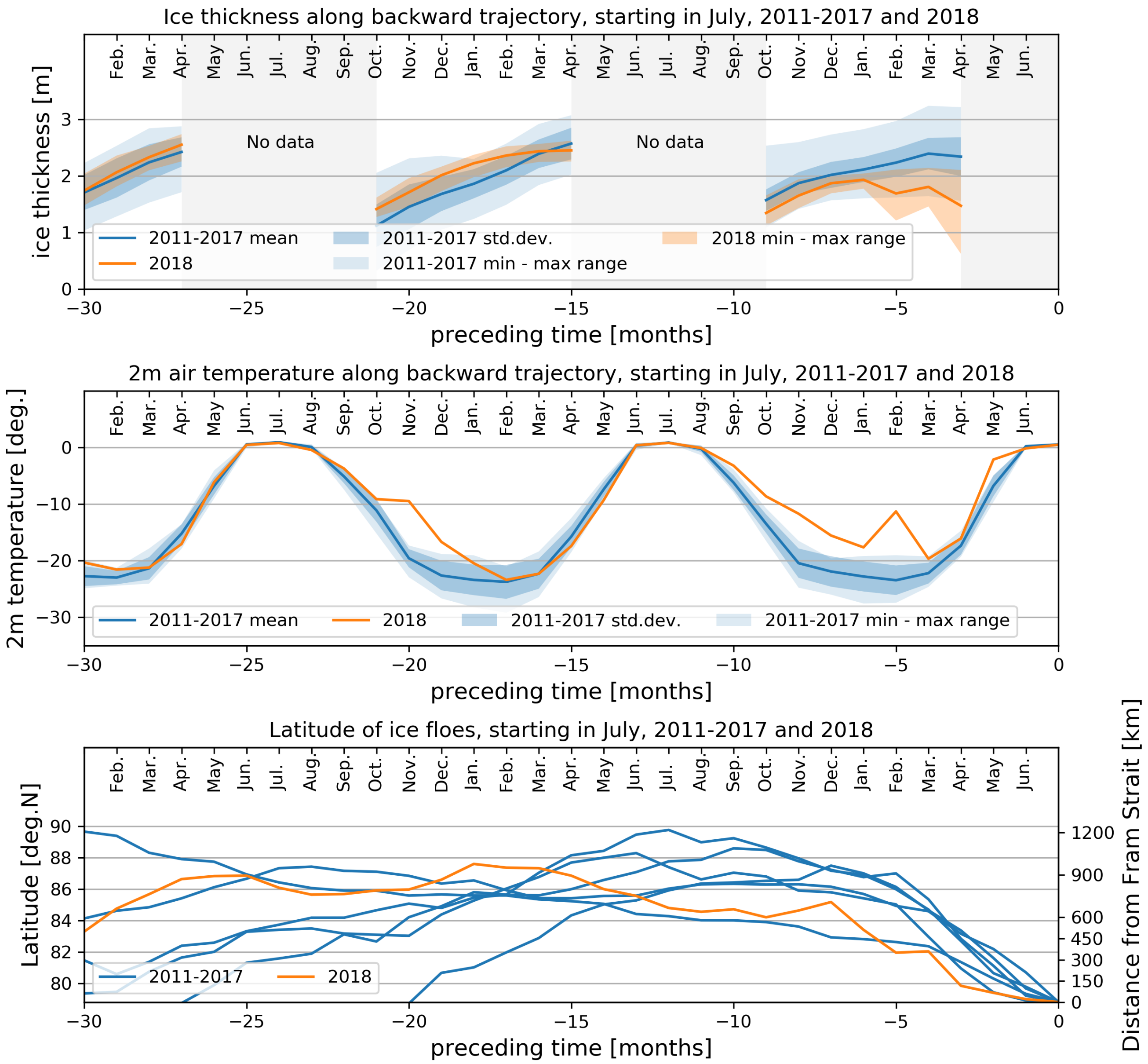

Figure S6. The same with Figure 5b, c, and d, but for reconstructions for ice floes arrived at Fram Strait in July, 2011 – 2017 and 2018.

Table S1. Mean ice floe positions and thickness before arrival in Fram Strait (2011 - 2017)

Arrival in Fram Strait	Latitude [°N]		Ice thickness [m]			Ice thickness change [m]	
	Two months before arrival	One month before arrival	Two months before arrival	One month before arrival	In Fram Strait	Preceding two months	Preceding one month
Nov.	82.6	81.4	---	1.30	1.17	---	-0.13
Dec.	83.6	81.7	1.47	1.60	1.30	-0.17	-0.30
Jan.	83.3	81.3	1.77	1.68	1.45	-0.32	-0.23
Feb.	82.7	80.7	1.87	1.75	1.53	-0.34	-0.22
Mar.	82.9	81.3	2.01	1.95	1.58	-0.43	-0.37
Apr.	83.9	81.9	2.22	2.29	1.63	-0.59	-0.66
May	83.4	80.8	2.46	2.18	---	---	---
Jun.	81.8	80.1	2.34	---	---	---	---

The positions of ice floes are estimated from backward trajectory analysis, while ice thickness estimates in each location in each month are obtained from averages of three ice thickness products derived from CS2 and SMOS (AWI, University Bristol and NERC CPOM). See Method section for description.

Table S2. Mean melt rate of ice floes for one month before arrival at Fram Strait (2011 - 2017)

Arrival in Fram Strait	Mean meltelt rate [cm day^{-1}]
Nov.	0.43
Dec.	0.97
Jan.	0.77
Feb.	0.73
Mar.	1.2
Apr.	2.2

The melt rate is calculated from ice thickness change along the trajectories of ice floes.

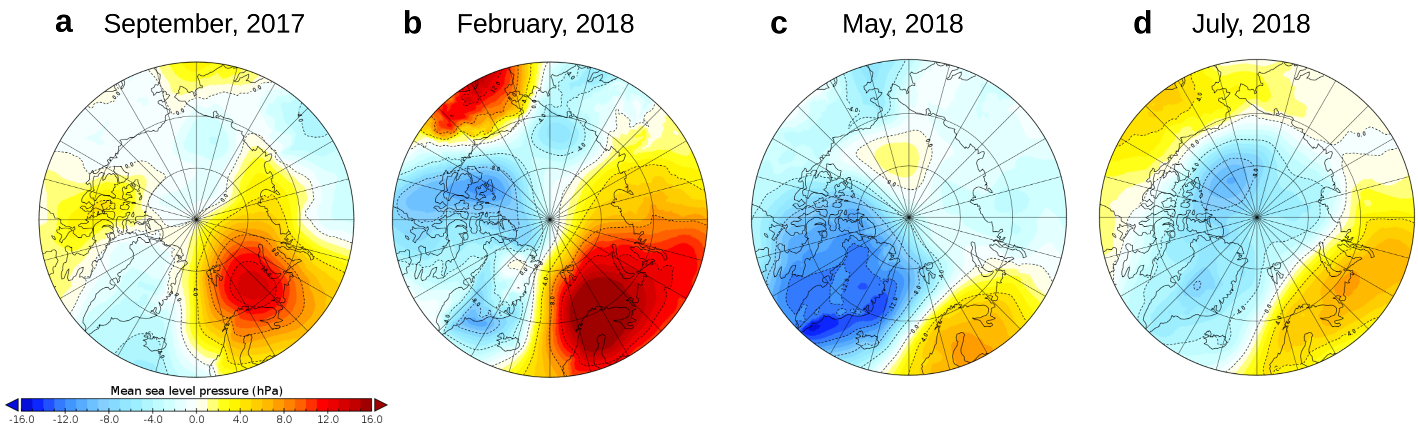

Figure S7. Spatial patterns of four SLP anomaly events from 2017 autumn to 2018 summer in ERA5: (a) September 2017, (b) February 2018, (c) May 2018, and (d) July 2018. The anomalies are defined by difference of monthly mean values from 1980 – 2018 monthly mean climatology.

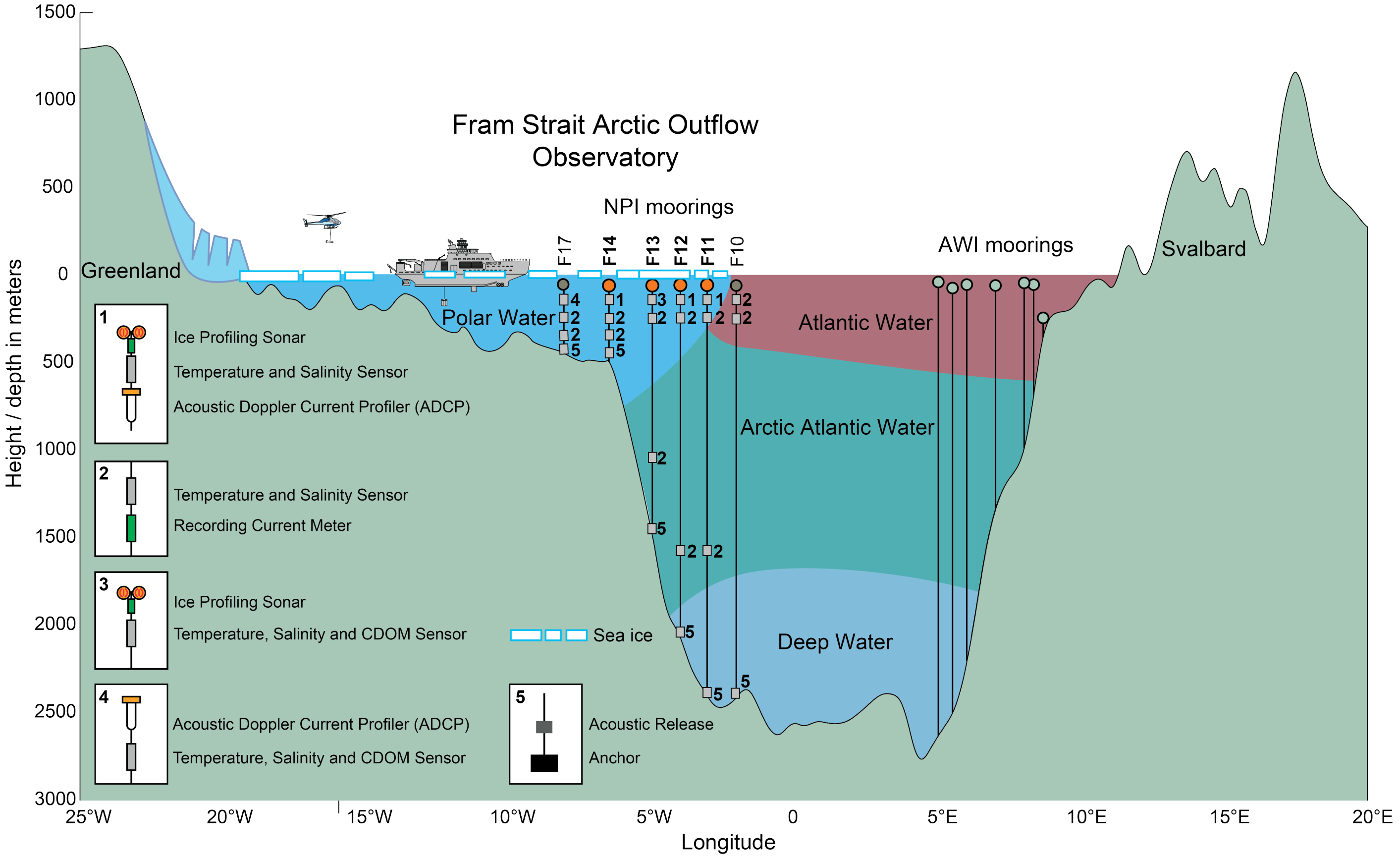

Figure S8. Sketch of the Fram Strait Arctic Outflow Observatory in the East Greenland Current in Fram Strait. ULS/IPSs were implemented at the top of F11 - F14 moorings. Figure credits: A. Igesund, P. A. Dodd (Norwegian Polar Institute).

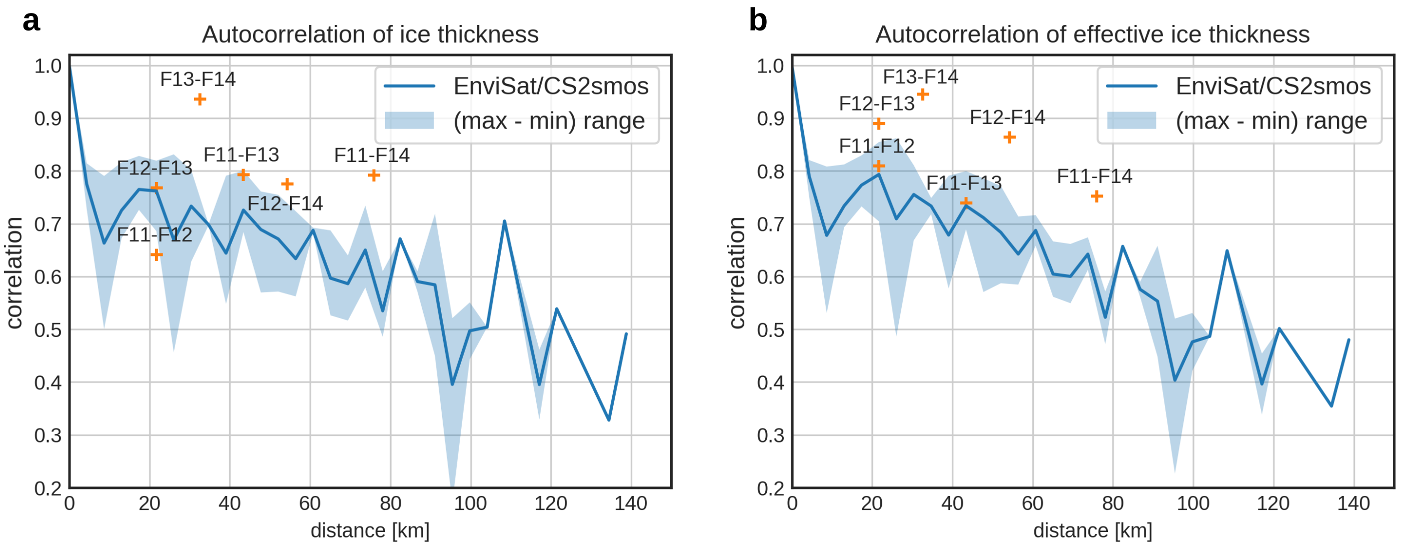

Figure S9. Autocorrelation of (a) ice thickness and (b) effective ice thickness at the Fram Strait zonal section calculated from combinations of ULS/IPSs (orange plus markers) and EnviSat/CS2smos (blue line). The correlations are calculated from the monthly mean ice thickness time series from 1990 to 2018 for ULS/IPS, while from 2002 to 2018 for ULS/IPSs. Note that the correlations of EnviSat/CS2smos are obtained from freezing months (Oct. - Apr.). The AWI product is used for the CS2smos correlation.

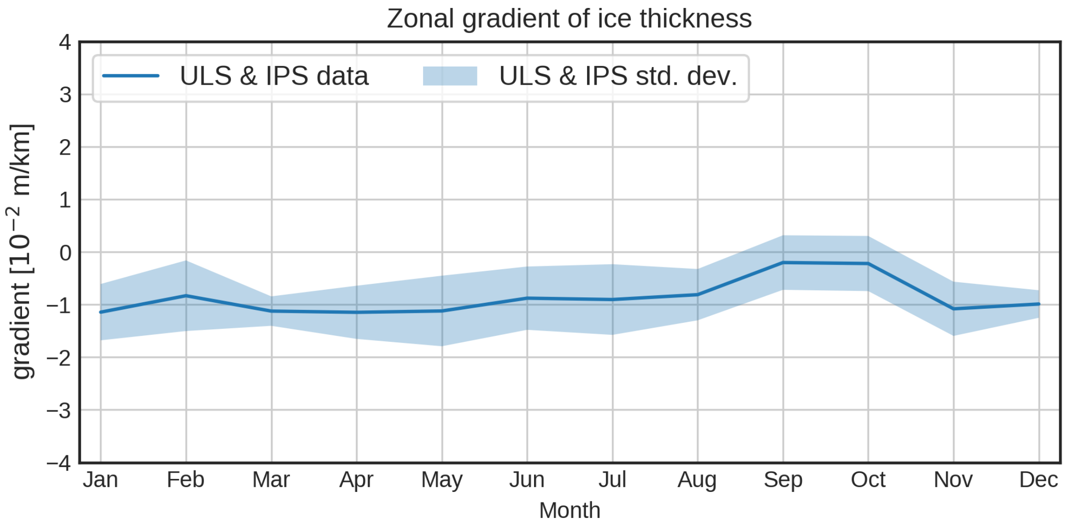

Figure S10. Seasonal cycle of zonal gradient of ice thickness in Fram Strait. The gradient is calculated from ice thickness difference between western and eastern most sites during the period of 2010 – 2018. The monthly mean values of the thickness are used to calculate the gradient.

Table S3. Uncertainty of monthly mean sea ice thickness at the Fram Strait section

Period [year]	instruments	Accuracy* [m]	Sampling interval [sec]	Uncertainty of monthly mean ice thickness [m]
1990 – 1991	ES300	0.2	480	2.7×10^{-3}
1991 – 2003	ES300	0.2	240	1.9×10^{-3}
2003 – 2006	ES300	0.2	120	1.3×10^{-3}
2007 – 2018	IPS4, 5	0.1	2	0.088×10^{-3}

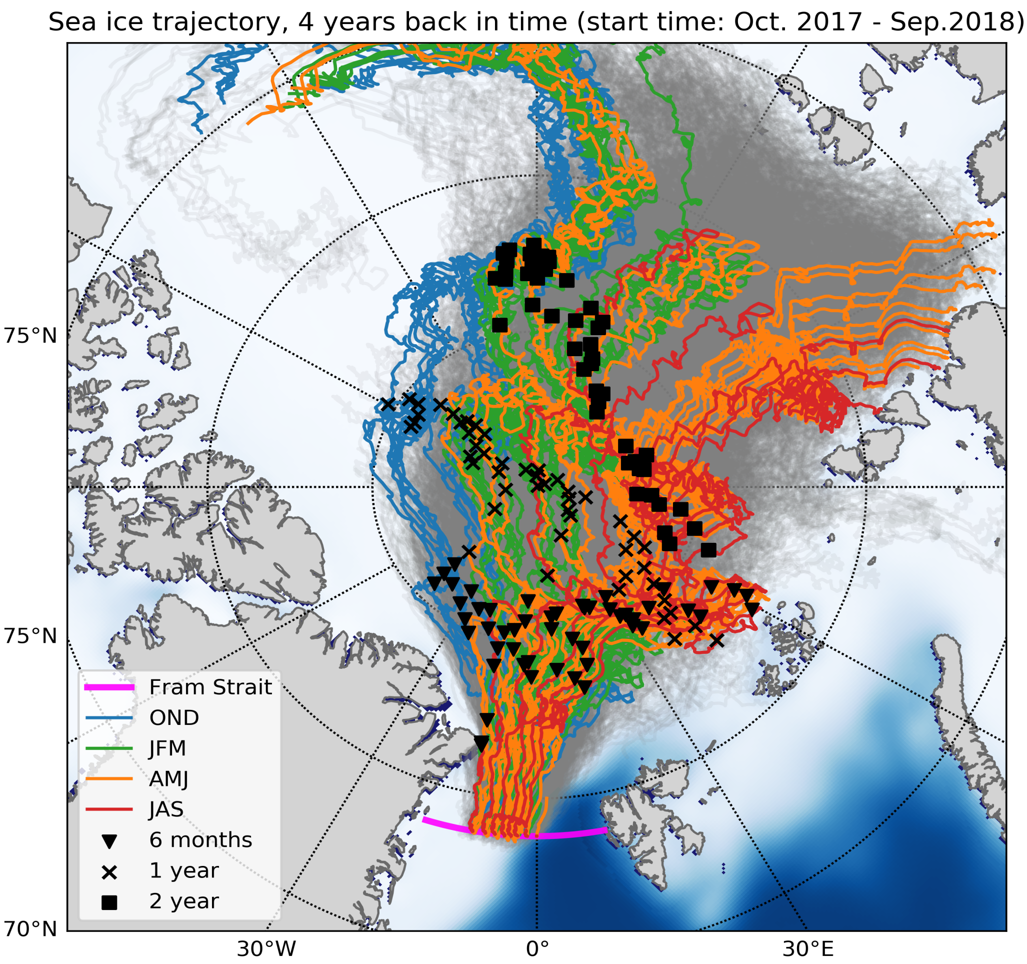

Figure S11. An example of sea ice trajectories of pseudo ice floes which arrived in Fram Strait from October 2017 to September 2018. Blue, green, orange and red lines correspond to pseudo ice floes arrived in Fram Strait in October, November, December (OND), January, February, March (JFM), April, May, June (AMJ), and July, August, September (JAS), respectively. The back markers (triangles, crosses, squares) denote positions of the floes 6 months, 1 year and 2 years before arrival at Fram Strait. The gray thin lines show all trajectories from 1990 to 2018. Note that the number of JAS trajectories is much less than other seasons because the trajectory calculation cannot be initiated due to low sea ice concentration in Fram Strait in 2018 summer.

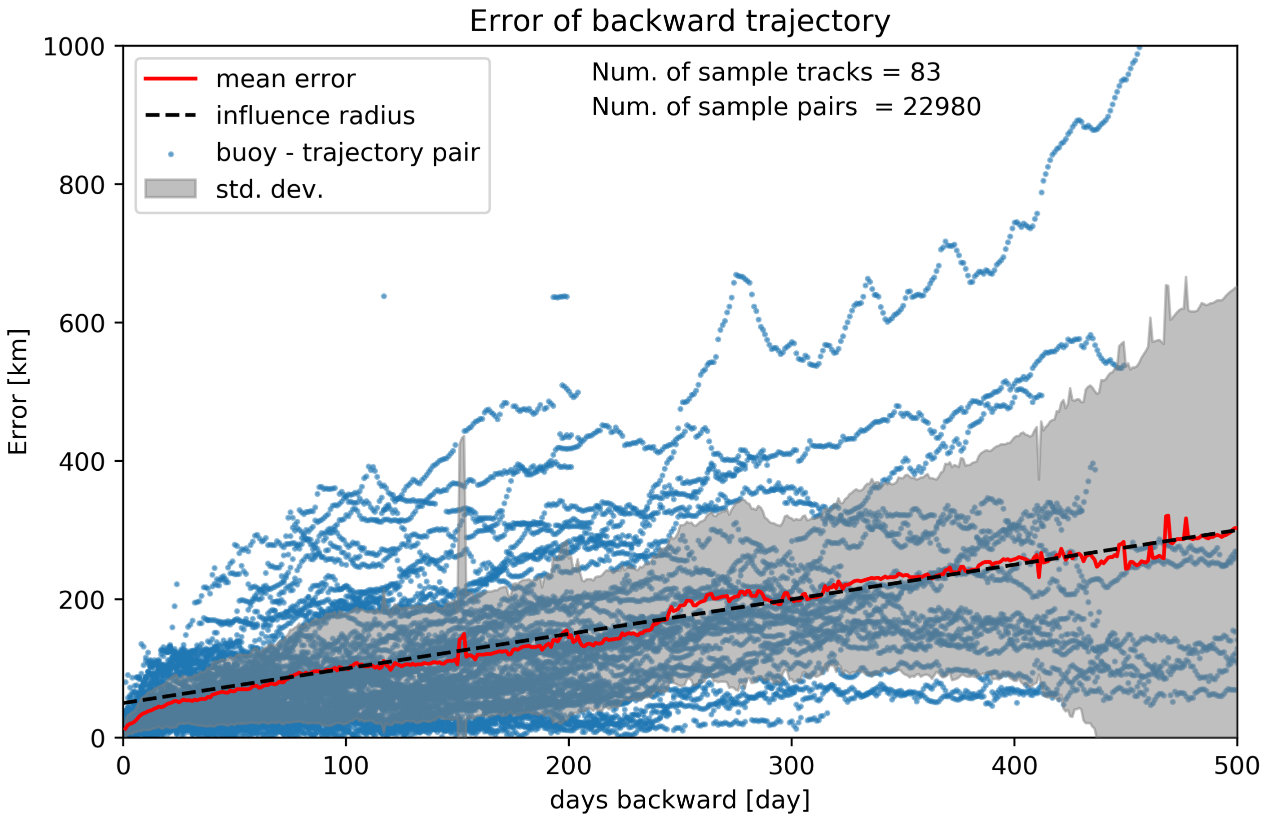

Figure S12. Evolution of position errors of backward trajectory calculation. Trajectories of 83 pseudo buoys initially located in Fram Strait are calculated by NSIDCv4 daily sea ice motion vectors backward in time. The pseudo buoy trajectories are compared with tracks of real buoys to calculate the position errors. The real buoy data were selected from International Arctic Buoy Program (83 buoy tracks which arrived in Fram Strait in the period from 2010 to 2018). The dashed black line is a fitted linear function which is used as an influence radius when calculating physical properties (2m air temperature and ice thickness) along the backward trajectories.

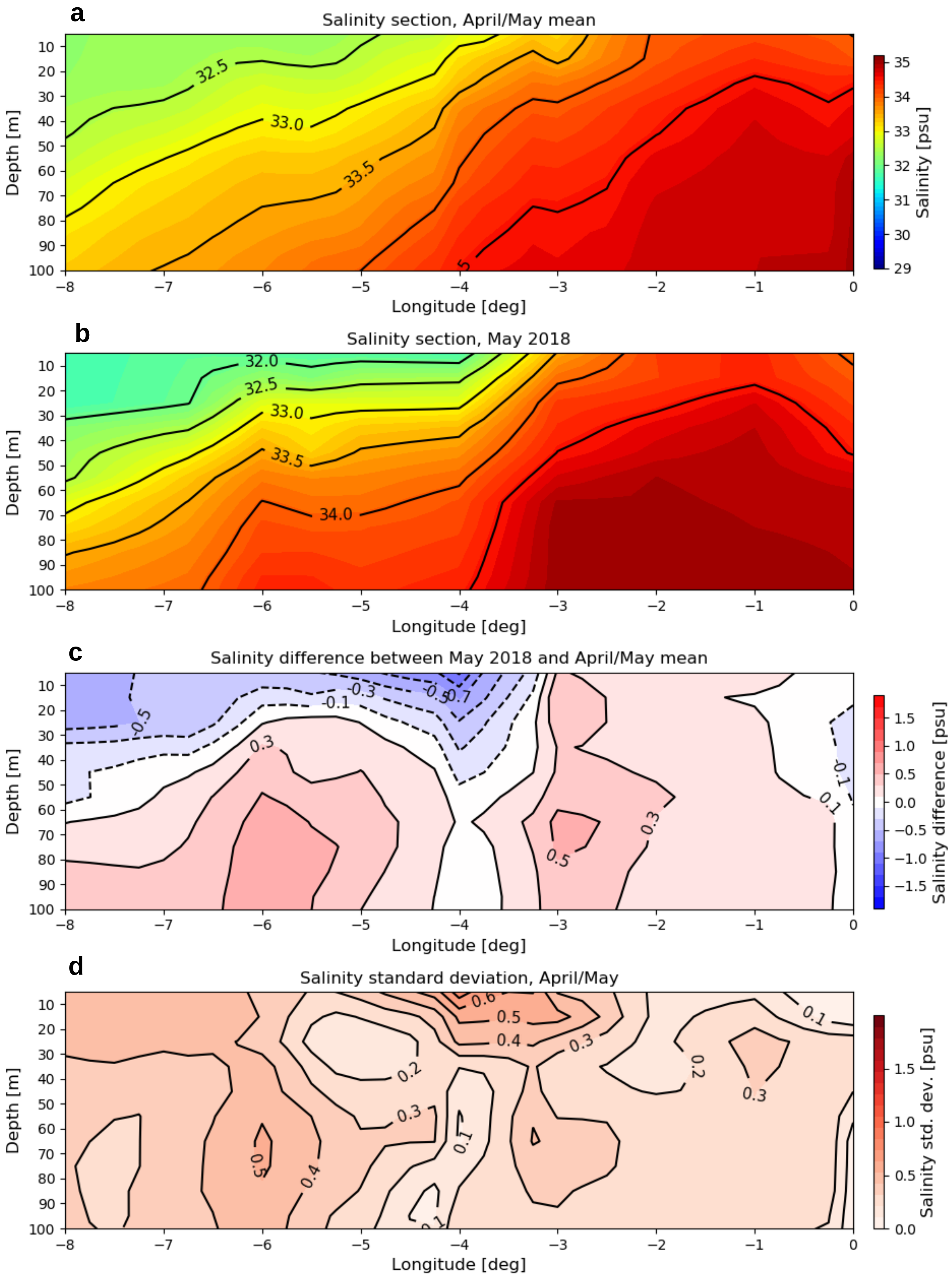

Figure S13. Salinity section at top 100 m in the western part of Fram Srait in Spring: (a) mean of available 5 years (2002, 2005, 2007, 2008 and 2018), (b) May 2018, (c) salinity anomaly in 2018 relative to the mean, and (d) salinity standard deviation calculated from the available 5 years data.

Table S4. Salinity anomalies in the shallow part of EGC (3 – 8° W) in May 2018.

Depth range	Salinity anomaly in 2018 [psu]	Salinity anomaly potentially caused by the 2018 ice reduction [psu]*
0 – 30 m	- 0.35	- 1.21
0 – 70 m	-0.01	-0.51

* The potential anomalies were calculated from the temporal mean stratification (Fig. R1 (a)) averaged in 3 – 8° W range in April/May plus virtual melt water input corresponding to the observed ice thickness anomaly of -1.22 m in May 2018.

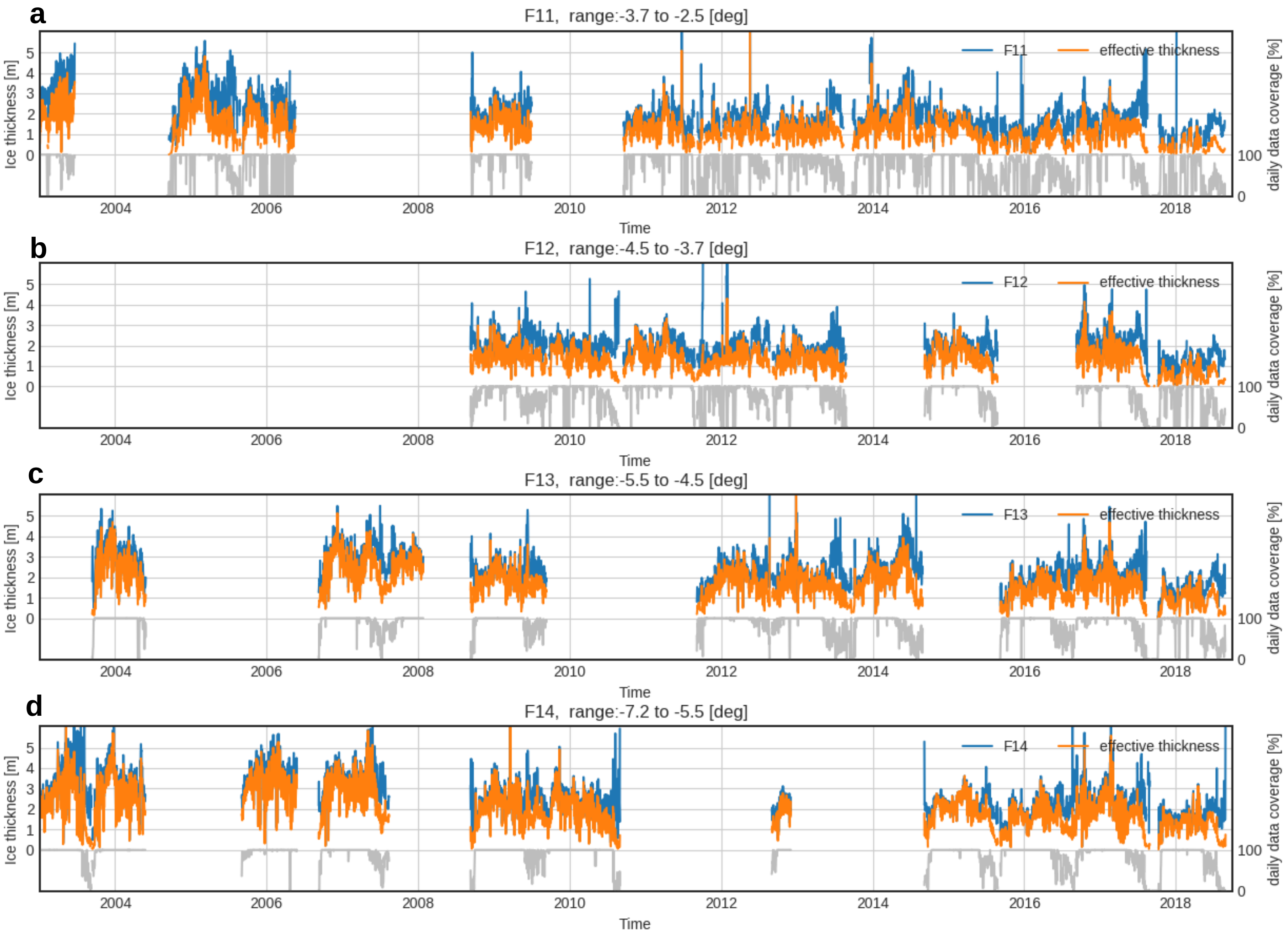

Figure S14. Time series of daily mean sea ice thickness and effective ice thickness at (a) F11, (b) F12, (c) F13, and (d) F14 from 2003 to 2018.
